# Use of Psychotropic Drugs among Children and Adolescents with Autism Spectrum Disorders in Denmark: A Nationwide Drug Utilization Study

**DOI:** 10.3390/jcm7100339

**Published:** 2018-10-10

**Authors:** Lotte Rasmussen, Niels Bilenberg, Martin Thomsen Ernst, Sidsel Abitz Boysen, Anton Pottegård

**Affiliations:** 1 Clinical Pharmacology and Pharmacy, Department of Public Health, University of Southern Denmark, 5000 Odense C, Denmark; lorasmussen@health.sdu.dk (L.R.); mternst@health.sdu.dk (M.T.E.); Sidsel.abitz@gmail.com (S.A.B.); 2 Child and Adolescent Psychiatric Department, Mental Health Hospital and University Clinic, Region of Southern Denmark, 5000 Odense C, Denmark; Niels.Bilenberg@rsyd.dk; 3 Odense Patient Data Explorative Network (OPEN), Odense University Hospital, 5000 Odense C, Denmark

**Keywords:** autism spectrum disorder, child, adolescent, psychotropic drugs, central nervous system stimulants, drug utilization, prevalence, Denmark

## Abstract

Children with autism spectrum disorder (ASD) have a considerable use of psychotropics. Leveraging nationwide registry data, we aimed to describe the use of psychotropics among children and adolescents with ASD in Denmark. Use of melatonin and attention-deficit/hyperactivity disorder (ADHD) medication increased from 2010 to 2017, while there were limited changes in use of antidepressants and antipsychotics. Thirty percent of the identified children used psychotropics in 2017 most commonly ADHD medication (17%) and melatonin (13%). Methylphenidate, sertraline and risperidone were most often prescribed. Most children filled more than one prescription and, across drug classes, at least 38% received treatment two years after treatment initiation. Use of psychotropics followed psychiatric comorbidities. Comorbidities did not affect age at treatment initiation. Use of psychotropics varied according to age and sex with limited use in the youngest children. In summary, psychotropic drug use has increased in children with ASD mainly due to an increase in the use of ADHD medication and melatonin. In accordance with previous studies, use seems to follow comorbidities. The long treatment duration underlines the need to investigate long-term effects of psychotropic drug use in children with ASD.

## 1. Introduction

Autism spectrum disorder (ASD) is a neurodevelopmental disorder with onset in childhood characterized by impaired social communication and interaction and unusually restricted, repetitive patterns of behaviour and interests [1,2]. It is estimated to affect 0.6% of individuals globally [3], although estimates up to 1.5% have recently been reported [4], also in Denmark [5].

Patients with ASD have a considerable use of psychotropic drugs, most commonly antipsychotics, attention-deficit/hyperactivity disorder (ADHD) medication and antidepressants [6]. However, no pharmacological agent has proven to be effective against the core symptoms of ASD [7,8]. As such, the pharmacological treatment of ASD mainly targets comorbid symptoms associated with ASD such as irritability and psychiatric comorbidities such as ADHD and obsessive-compulsive disorder (OCD) [7,8]. With the exception of a few antipsychotics approved for the treatment of severe behavioural problems (irritability) in ASD [9], psychotropic drugs are used off-label when used to treat the core symptoms of ASD. However, previous studies have demonstrated non-negligible use of psychotropic drugs in children with ASD who had no psychiatric comorbidity [10,11]. Considering the limited evidence of efficacy and safety for the majority of psychotropic drugs in ASD populations [9,12] this raises concerns about the rationality in drug use. 

In this study, we aimed to describe the use of ADHD medication, antidepressants, antipsychotics and melatonin in children and adolescents with ASD in Denmark. More specifically, we wanted to explore changes in prevalence of medication use over time and the duration of drug use. Based on the most recent data, we further explored how medication use is affected by age, sex and psychiatric comorbidities. 

## 2. Experimental Section

Using the Danish nationwide health registries, we identified all children and adolescents born between 1992 and 2011 with a hospital diagnosis of ASD after the age of three and before the age of 18. We extracted data on all their psychotropic prescriptions and psychiatric hospital diagnoses from birth and until the end of 2017.

### 2.1. Data Sources

We used the Danish National Patient Registry [13] to identify children and adolescents with a psychiatric disorder coded under the F-code of the International classification of diseases (ICD)-10 system. We used data on psychiatric diagnoses since 1995 and until 31 December 2017.

The Danish Psychiatric Central Register [14] contains information on all patients treated at psychiatric departments in Denmark since 1969 and onwards. Since 1995 it has included data on outpatient treatment and emergency room contacts. Data includes date of admission and discharge, or start and end date of outpatient and emergency room contacts and related diagnoses. From 1995 and onwards the Danish Psychiatric Central Register has been an integrated part of the Danish National Patient Register.

We used the Danish National Prescription Registry [15] to extract information on psychotropic prescriptions filled by our study population at community pharmacies from the date of birth and until 31 December 2017. We extracted information on dispensing date and the anatomical therapeutic chemical classification (ATC) code of the dispensed medication.

By using the Danish Civil Registration system [16] and the unique personal identification number, we were able to link information on diagnoses with prescriptions. Information on death and migration was extracted from the Danish Civil Registration system.

### 2.2. Study Population

In this study, we used registry data and ICD-10 classification to define a cohort of children and adolescents in Denmark born between 1992 and 2011 with a hospital diagnosis of ASD before 31 December 2017. We disregarded children moving to Denmark during the study period as we did not have full data coverage on these children. ASD was defined by the following ICD-10 codes: F84.0 (childhood autism), F84.1 (atypical autism), F84.5 (Asperger’s syndrome) and F84.8 (other pervasive developmental disorders). We disregarded children who only had an unspecified ASD diagnosis (F84.9) based on clinical knowledge about diagnostic practice in Denmark. Due to diagnostic uncertainties in early life [17], we required that the ASD diagnosis was established or confirmed at or after the age of 3 and before the age of 18. If a child had more than one ASD diagnosis we used the date of the first ASD diagnosis to assign age at first diagnosis (see below) also if this was before the age of three. Of note, while pervasive developmental disorders and hyperkinetic disorders (ADHD) are mutually excluding each other according to ICD-10, the Danish Child and Adolescent Psychiatric Society have since 2008 recommended that both diagnoses should be registered in the same patient as comorbid diagnoses. Before 2005, the use of standardized diagnostic tools was limited in Denmark. After 2005, assessment using Autism Diagnostic Observation Schedule (ADOS) has become routine, in some clinics supplied with Autism Diagnostic Interview (ADI).

All children in the cohort were followed in the health care registries from the date of birth and until censoring at their 18th birthday, date of death/emigration, or at 31 December 2017 (end of study period), whichever came first.

The study population was categorized according to the age they turned in a given calendar year as 3–5 years, 6–11 years and 12–17 years.

### 2.3. Study Drugs

We focused on the use of ADHD medication (ATC: N06BA), antidepressants (N06A), antipsychotics (N05A) and melatonin (N05CH01). Melatonin has been incompletely registered in the Danish National Prescription Registry until 2012. Therefore, melatonin prescriptions were only included from 2012 and onwards. Besides describing use of the four individual drug classes, we defined a group of “any” medication use and a group of “any without melatonin”, defined as use of ADHD medication, antidepressants, or antipsychotics with or without (w/o) melatonin.

### 2.4. Analysis

Descriptive analyses were performed to describe the use of ADHD medication, antidepressants, antipsychotics and melatonin in our cohort of children and adolescents with ASD over time from 2010 to 2017 with more detailed analyses focusing on data from 2017.

For analyses of data from the calendar year 2017, we restricted our study population to children and adolescents with ASD 6–17 years old in 2017, that is, children with ASD born between 2000 and 2011 (see Appendix A) and to those who were still present in the cohort at 1 January 2017, that is, not censored prior to 2017.

#### 2.4.1. Characteristics of Study Population

We described our entire study population of children with ASD born between 1992 and 2011 and the specific cohort of children with ASD in 2017. They were described according to ASD diagnosis subtype based on the most recent ASD diagnosis before end of follow up, median age at first ASD diagnosis according to the first subtype, age distribution in 2017 (for the cohort of children with ASD in 2017), sex, the top ten most frequent psychiatric comorbidities from birth and until end of follow-up and finally selected psychiatric comorbidities from birth and until end of follow-up. The comorbidities investigated were ADHD, intellectual disability, and other psychiatric comorbidities (besides ADHD and intellectual disability). For a detailed description of how we defined these comorbidities, see Appendix B.

#### 2.4.2. One-Year Prevalence Proportion of Medication Use from 2010 to 2017

To investigate how use of the four drug classes has changed over time, we estimated the one-year prevalence proportion for the use of ADHD medication, antidepressants and antipsychotics in our entire study population from 2010 to 2017 and from 2012 to 2017 for melatonin. For each calendar year, the numerator was the number of children filling ≥2 prescriptions within the given year and the denominator was the number of children with ASD who were not censored prior to the given year. The analysis was stratified by age. The criterion of ≥2 prescriptions as opposed to ≥1 prescription was used to limit the influence from trial use or sporadic use of drugs and was subject to a sensitivity analysis (see below).

#### 2.4.3. Early Discontinuation and Persistence Rate

To investigate the duration of use of the four drug classes, we described the rate of early discontinuation and persistence to treatment. First, as a measure of early discontinuation, we estimated the proportion of children and adolescents who filled a second prescription within 180 days after the first ever prescription of ADHD medication, antidepressants, antipsychotics and melatonin. The numerator was the number of children who filled a second prescription and who were not censored within the first 180 days. The denominator was the number of children who started the medication and who were not censored within the first 180 days. Second, for users of ADHD medication, antidepressants, antipsychotics and melatonin, we estimated the proportion of children who filled a prescription within 3 months before day 180, 365 and 730 where day 0 marked the date of initiation. The numerator was the number of children having filled a prescription of the given drug within 3 months before day 180, 365, or 730 and who were not censored at that specific day. The denominator was the number of children who started the medication and who were not censored at that specific day. This approach is a modified version of the proportion of patients covered (PPC) method used to describe treatment duration and persistence [18]. The analysis was stratified by age and restricted to children initiating treatment between 2010 and 2015. In supplementary analyses, we repeated this analysis for children having ASD and comorbid ADHD and for children initiating treatment in 2000–2004 and 2005–2009, respectively.

#### 2.4.4. Age and Sex Stratified Prevalence Proportion of Medication Use in 2017 

To investigate if use of the four drug classes is affected by age and sex, we estimated the age and sex stratified prevalence proportion for the use of the four drug classes in 2017. The numerator was the number of children filling ≥2 prescriptions at a given age and sex and the denominator was the number of children with ASD at the same age and sex. 

#### 2.4.5. Age-Stratified Prevalence Proportion of Medication Use by Birth Year

To investigate whether use of the four drug classes is affected by year of birth, we estimated the age-stratified prevalence proportion for the use of ADHD medication, antidepressants, antipsychotics and melatonin according to birth year. The numerator was the number of children filling ≥2 prescriptions at a given age and the denominator was the number of children with ASD at the same age who was not censored prior to the year where they turned the given age. The analysis was restricted to children with ASD born between 2000 and 2011.

#### 2.4.6. Drug Use According to Psychiatric Comorbidities 

To describe the prevalence of the use of the four drug classes in 2017, we calculated the proportion of children filling ≥2 prescriptions of ADHD medication, antidepressants, antipsychotics or melatonin in 2017. Furthermore, to investigate whether medication use in 2017 is affected by psychiatric comorbidities, this analysis was stratified by the presence of selected comorbidities as described above. In an exploratory analysis, we estimated the prevalence of the ten most common “other” psychiatric comorbidities for children and adolescents included in this analysis who were identified with “other” psychiatric comorbidities.

#### 2.4.7. Age at First ASD Diagnosis and First Prescription

To investigate at what age children and adolescents with ASD initiate treatment, we estimated the age at first ASD diagnosis and the age at first ever prescription of ADHD medication, antidepressants, antipsychotics and melatonin for those with ≥2 prescriptions filled. This analysis was stratified by presence of selected comorbidities as described above.

#### 2.4.8. Most Commonly Used Drugs in 2017 

To identify the specific drugs used, we identified the five most commonly prescribed medications on the fifth ATC level within each of the three drug classes in 2017 (disregarding melatonin). For each medication identified, we calculated the proportion of the total number of prescriptions filled in 2017 within that drug class. The analysis was stratified by age.

#### 2.4.9. Sensitivity Analyses

Due to potential differences in medication use between children receiving an ASD diagnosis early versus late in childhood, we performed a sensitivity analysis restricting our analysis to children having received an ASD diagnosis before the age of seven. Further, we changed our definition of prevalent medication use from having filled at least two prescriptions within a year to having filled at least one prescription within a year. 

### 2.5. Statistical Analysis

Descriptive statistics including medians with interquartile ranges (IQR) and frequencies were reported. All calculations were performed using STATA Release 15.1 (Stata Corp, College Station, TX, USA).

### 2.6. Ethical Approval

This study was approved by the Danish Data Protection Agency. According to Danish law, purely registry-based studies does not need approval from an Ethics Committee [19].

## 3. Results

Our study population comprised 23,935 Danish children and adolescents born in 1992–2011 with an ASD diagnosis. The median age at first ASD diagnosis was 9 years (IQR: 6–13), 75% were male, 31% had comorbid ADHD and 15% had comorbid intellectual disability (Table 1). A total of 14,210 children 6–17 years old had ASD in 2017 (4850 6–11 years old and 9360 12–17 years old). The median age for these children in 2017 was 13 years (IQR: 10–16). Comparing females and males with ASD, males more often had ADHD (F/M: 29%/36%) and females more often had “reaction to severe stress and adjustment disorders” (F/M: 21%/11%), “depressive episode” (F/M: 10%/3%) and “other anxiety disorders” (F/M: 9%/4%). In addition, females had a slightly higher median age at first ASD diagnosis both overall (F: 10 (IQR: 6–13); M: 8 (IQR: 5–11)) and in 2017 (F: 14 (IQR: 11–16); M: 13 (IQR: 10–15)) compared to males.

**Figure 1:**
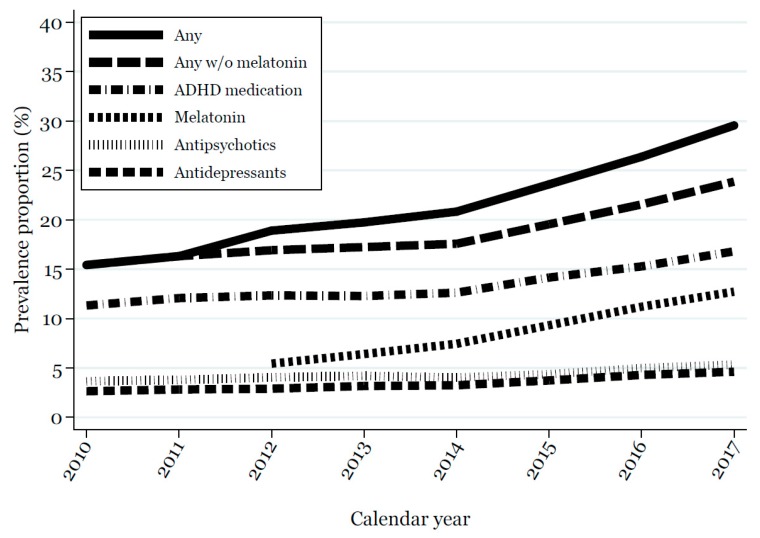
One-year prevalence proportion for the use (≥2 prescriptions) of attention-deficit/hyperactivity disorder (ADHD) medication, antidepressants, and antipsychotics from 2010 to 2017 and melatonin from 2012 to 2017 in children and adolescents 3–17 years old with autism spectrum disorder (ASD).

Use of melatonin increased from 2012 to 2017 (Figure 1), while the use of ADHD medication increased from 2014 to 2017 (Figure 1), mainly due to an increase in use among children 6–11 years old (Figure S1). Conversely, the one-year prevalence proportion for the use of antipsychotics and antidepressants was reasonably stable from 2010 to 2017, both overall (Figure 1) and when looking at single age groups (Figure S1).

**Figure 2:**
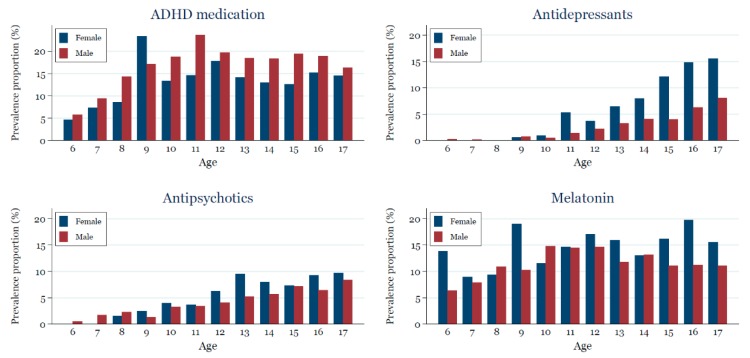
Age-stratified prevalence proportion for the use of ADHD medication, antidepressants, antipsychotics, and melatonin in 2017 for children and adolescents 6–17 years old with ASD.

Use of the four drug classes varied by sex and age (Figure 2). Except for ADHD medication, the prevalence of use was in general higher among females than males. In a *post hoc* analysis, the higher use of antidepressants among females persisted when stratifying by comorbidity, whereas the slightly increased use of antipsychotics for females compared to males was primarily seen among those without ADHD (e.g., those with intellectual disability and other psychiatric comorbidities). Use increased with age for antidepressants and for antipsychotics, while the pattern was less clear for ADHD medication and melatonin. Except for melatonin where older birth cohorts had higher use than younger birth cohorts, the prevalence of use was not affected by year of birth (Figure S2).

The rate of early treatment discontinuation was lowest for use of ADHD medication where ≤7% failed to fill a second prescription within 180 days. Few users of antidepressants and antipsychotics (*n* ≤ 30) were 3–5 years old which prohibited meaningful comparison for this age group. However, except for ADHD medication and antidepressants, where the rate of early discontinuation was almost similar between age groups, the rate was slightly higher among adolescents. Persistence to treatment was highest for use of ADHD medication and in general varied between 50–82% after 180 days, 43–77% after one year and 38–73% after two years. For use of ADHD medication and melatonin adolescents had lower persistence rates (Table 2). Restricting to children with comorbid ADHD gave slightly higher persistence rates for ADHD medication, antipsychotics and melatonin in the youngest, while giving slightly lower persistence rates for antidepressants (Table S1). Low number of users in some study years (*n* ≤ 30) limited comparability between study years (Table S2).

In 2017, 30% of children and adolescents (6–17 years of age) with ASD used at least one of the four drug classes. The one-year prevalence proportion for the use of ADHD medication, antidepressants, antipsychotics and melatonin was 17%, 5%, 5% and 13%, respectively. Changing our criterion of two prescriptions to one prescription only caused small changes to the prevalence in 2017; 33% used at least one of the four drug classes, 18% used ADHD medication, 5% used antidepressants, 6% used antipsychotics and 17% used melatonin (Figure S3).

**Figure 3:**
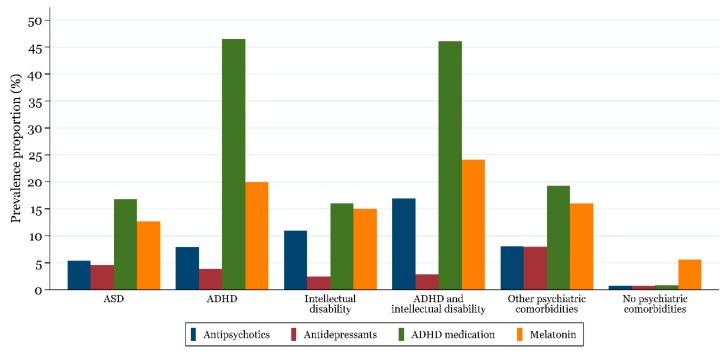
One-year prevalence proportion for the use of ADHD medication, antidepressants, antipsychotics, and melatonin in 2017 in children and adolescents 6–17 years old with ASD by psychiatric comorbidity.

Use varied according to psychiatric comorbidity and children with comorbid ADHD and intellectual disability was most commonly medicated (60%). Children with ASD and no psychiatric comorbidity were least frequently medicated (7%), mainly driven by use of melatonin (6%) (Figure 3).

In 2017, the single drug substances most often prescribed were methylphenidate (6–11 years old: 68% of all ADHD medications; 12–17 years old: 61%), risperidone (6–11 years old: 76% of all antipsychotics; 12–17 years old: 43%) and sertraline (6–11 years old: 76% of all antidepressants; 12–17 years old: 74%) (Table 3).

The age at first ASD diagnosis varied slightly according to psychiatric comorbidity. Children with comorbid intellectual disability were diagnosed at a younger age (5 years; IQR: 4–8) compared to children with other comorbidities (9 years; IQR 6–12). The age at first prescription of the four drug classes was generally not affected by psychiatric comorbidity. Across all comorbidities, ADHD medication was started at a younger age than antidepressants (9 years versus 14 years) (Table S3).

The most common psychiatric diagnoses in children with ASD and other psychiatric comorbidities were “reaction to severe stress and adjustment disorders” (26%) and “other behavioural and emotional disorders with onset usually occurring in childhood and adolescence” (22%) (Table S4).

Children who received the ASD diagnosis before the age of seven more often had intellectual disability (28%) and were younger (median age in 2017: 11 years; IQR: 8–14). While they on average started medication use at a younger age, they used less psychotropics (in 2017, 23% used at least one of the four drug classes compared to 30% in the main analysis), in particular antidepressants (1% compared to 5% in the main analysis). Further, the use of ADHD medication was lower specifically among females.

## 4. Discussion

From this nationwide study of the use of psychotropic drugs among children and adolescents with ASD in Denmark, several findings deserve to be highlighted. First, the overall rate of psychotropic drug use in Danish children and adolescents with ASD increased from 2010 to 2017, however solely driven by an increase in the use of ADHD medication and melatonin. Second, most children filled more than one prescription and, across drug classes, more than 38% of patients received treatment after two years, suggesting that in many children there is a long–term need for psychotropic drugs. Third, psychotropic drug use followed comorbidities and the drugs used was generally in accordance with guidelines. Finally, psychotropic drug use varied by sex and age, likely reflecting age and sex differences in comorbidities.

Our study has several strengths. First, our study was based on data from high quality registries [13,14,15] and allowed us to describe utilization patterns in an unselected group of patients with a hospital diagnosis of ASD. Second, the ASD diagnoses used in this study has been validated with good results, although there might be slight misclassification of ASD according to ICD-10 pervasive developmental disorder subtypes [17], which might attenuate observed differences between subtypes.

Our study also has some limitations. First, the indication for drug use was not available in the registries. Thus, we do not know for which indication the drug was prescribed. Second, as our study relies on dispensed prescriptions we do not know whether the drugs were actually consumed by the patients. However, our criterion of filling at least two prescriptions should limit the influence from this. Third, we included patients based on hospital diagnoses only. Thus, patients receiving an ASD diagnosis outside the hospital, for example, by private practicing specialists, are not included in our study; there is currently 16 private practicing child- and adolescent psychiatrists in Denmark (medcom.dk). This hold some implications in terms of generalizability, as patients with mild ASD might not be seen at the hospital. However, while the proportion of patients diagnosed with ASD outside the hospital is unknown, it is expected to be low. Our population is thus expected to represent the vast majority of children/adolescents with ASD in Denmark. Fourth, our category of “no comorbidity” was based on the absence of psychiatric hospital diagnoses. This category should be interpreted cautiously as patients might have a diagnosis from outside the hospital and/or might not have their comorbidity coded at the hospital. Lastly, we included patients who received an ASD diagnosis up until the end of 2017 meaning that we did not restrict the diagnosis to occur for some time before the end of the data. Thus, we might have included some ASD patients where the clinical diagnostic process was not yet complete, potentially resulting in tentative diagnoses not being removed from the registry upon diagnostic completion [17]. However, we only included final diagnoses of discharge (primary and secondary) (i.e., no referral diagnoses) which should limit this.

That 30% of Danish children and adolescents with ASD used psychotropic drugs in 2017 lies within the range reported in other studies for similar age groups, although there is considerable variation among studies (3–80%) [6]. Except for the use of ADHD medication, the use of antidepressants and antipsychotics found in Danish children with ASD is generally lower than the median prevalence of 17% for antipsychotics and 12–22% for antidepressants reported in a recent systematic review which was mainly based on studies from North America [6]. Besides cultural differences and differences in drug policies [6], this may be due to methodological differences in for example included drugs and the prevalence measures applied. However regarding the latter, we used one-year prevalence proportions which will yield higher results than measures of for example point-prevalence proportions or current use which was applied in some studies [6]. Our conservative criterion of requiring the filling of at least two prescriptions did not explain the lower prevalence as revealed by our sensitivity analysis. The prevalence of use in Denmark in 2010 is, however, in line with prevalence of 12.5% for ADHD medication and 3.8% for antidepressants reported in Germany in 2009, whereas the use of antipsychotics was substantially higher in Germany (11.7%) [20].

A few studies have described time trends in the use of psychotropic drugs in children with ASD with varying results and during different time periods [20,21,22,23]. The study with the most recent data and most comparable to ours found an increasing time trend from 2004 to 2009 in Germany [20]. In Denmark, the use of ADHD medication has been increasing both nationwide (2000–2010) [24] and in children with ASD (from 1999 to 2010) [25] and use of melatonin has also been increasing [26]. Thus, it is not unexpected that this is also reflected in children with ASD, although the increase in the use of ADHD medication in Denmark from 2010 to 2017 is limited compared to that observed in Germany from 2004 to 2009 [20].

Only a few studies have looked at treatment duration and persistence in children with ASD [10,21,22] and different methodological approaches hinders direct comparison to our results. One study showed that adolescents had a high likelihood of staying medicated over a 4.5 year period [22] which corresponds well with our finding that at least around 38% of children and adolescents received treatment after two years. Two studies based on US populations showed that number of days on treatment and length of treatment increased with higher age [10,21]. Our data, on the contrary, suggest that adolescent users of melatonin and ADHD medication are less persistent with their medication compared to children in line with similar findings in Danish children and adolescents using ADHD medication [27]. Higher persistence rate in children might be explained by greater parent involvement in controlling treatment in children with ASD compared to adolescents. The lower rate of early discontinuation and the higher persistence found for use of ADHD medication compared to antidepressants, antipsychotics and melatonin might be explained by the on- or off-label status of the drugs. Physicians may be more willing to prescribe and initiate ADHD medication compared to the other drug classes and they may use it less on a trial-and-error approach. Side effects or non-response may cause patients to discontinue treatment. The rate of early discontinuation found among children and adolescents with ASD for use of ADHD medication are less than the overall rate of 12.5% in 2012 found in a nationwide Danish study [28]. Although short-term use of methylphenidate has shown some benefit in children with ASD and comorbid ADHD [29], the response to treatment is generally thought to be lower compared to children with ADHD only [9,30] with one study reporting a response rate of 49% [31]. Furthermore, in children with ASD and comorbid ADHD, methylphenidate seems to be less tolerated with higher discontinuation rates due to side effects than in children with ADHD only [9,30,31]. Thus, the high persistence to this treatment is somewhat surprising and might warrant further study.

Similar to our study, previous studies [10,32] have shown that psychiatric comorbidities, especially ADHD, increases the need for psychotropic drugs. Our study indicates that other comorbidities such as for example those included under “reaction to severe stress and adjustment disorders” and intellectual disability also increases the need for psychotropic drugs, although to a lesser extent than ADHD. The use of psychotropic drugs in children with ASD in Denmark seems to follow the comorbidity and not being targeted against the ASD itself, for example ADHD medication in children with comorbid ADHD. This is reassuring considering that there is no effective treatment against ASD core symptoms and the fact that the majority of children with ASD have a comorbid psychiatric disorder. Contrary to our findings, one study showed a noticeable drug use in children with ASD and no comorbid disorder, potentially indicating that medication is used to treat ASD [10]. Although in Denmark melatonin is not approved for use in children, off-label use to treat for example sleep disturbances has been reported [33,34,35]. Melatonin has shown good effect in children with ASD [36] and the low use of melatonin in children with no reported comorbidity might therefore be explained by off-label use to treat sleep problems which often affects children with ASD [37].

Methylphenidate, sertraline and risperidone (and aripiprazole in adolescents) accounted for more than half of prescriptions in each of the three drug classes. In Denmark, methylphenidate is first-line treatment for ADHD, sertraline is first-line treatment for OCD and risperidone is the only antipsychotic approved for use in children ≥6 years with ASD and severe behavioural problems [38]. Thus, in Denmark use of psychotropic drugs in children with ASD generally seems to follow the guidelines available to treat comorbid disorders. Contrary to licensing status in for example the US, aripiprazole is not approved to treat irritability in children with ASD in Denmark. In adolescents, aripiprazole might therefore be used to treat schizophrenia for which it is indicated [38]. However, in both children and adolescents, it is possible that aripiprazole is used off-label (but with good evidence [9,39]) as a substitute for risperidone in patients with ASD where risperidone is not tolerated. Accordingly, two studies conducted in 2014 among Danish children in two different psychiatric settings showed that the off-label prescribing rate of aripiprazole was high mainly due to use for unapproved indications [34,35] with ASD appearing as one [34].

The low use of antipsychotics in Danish children with ASD is in line with recommendations of reserving use to children where the risk of side effects, for example metabolic side effects such as weight gain [40], are outweighed by the benefits [7,41]. There is limited evidence for the use of antidepressants to treat core symptoms of ASD [42]. Accordingly, use was limited among children with ASD only and highest among children with other psychiatric comorbidities with the main agents used being sertraline and fluoxetine. Although we cannot be certain of the indication for treatment, the prevalence of use of antidepressants is in line with the prevalence of OCD and depressive episodes (approved indications) in Danish children with ASD.

The variation observed according to age and sex is supported by other studies [6] and can most likely be explained by age and sex differences in comorbidity patterns [43,44]. Males more often present with ASD than females and further there seems to be a gender difference in how core symptoms of ASD presents, with males showing more externalizing symptoms such as aggressive behaviour and hyperactivity and females showing more internalizing symptoms such as anxiety and depression [45]. Accordingly, males tend to use more ADHD medication and antipsychotics [6], while females tend to use more antidepressants and anxiolytics [6]. The female to male ratio for the use of antipsychotics in Danish children ≥9 years with ASD is thus surprising. Females with ASD are often under-recognized compared to males [1] and often receive the diagnosis later [46]. Furthermore, it has been shown that behavioural problems affect the likelihood of receiving an ASD diagnosis differently in males and females [47]. A higher threshold for diagnosing ASD in females might lead to females with ASD in our study representing more severe or complex cases which could explain the slightly higher use of antipsychotics. Of note, the slightly higher median age among females might explain part of the higher use of antipsychotics. In addition, the higher proportion of for example “reaction to severe stress and adjustment disorders” supports our hypothesis of females with an ASD diagnosis on average being more severe cases. Similarly, the higher use of antidepressants could be attributed to females more often having emotional disorders compared to males.

## 5. Conclusions

In conclusion, the overall rate of psychotropic drug use in Danish children and adolescents with ASD increased from 2010 to 2017 solely driven by an increase in the use of ADHD medication and melatonin. In accordance with other studies, use varied according to psychiatric comorbidity, age and sex. Comorbidities are treated rather than the ASD itself and the drugs used are generally in accordance with guidelines to treat comorbidities. The long duration and low rate of early treatment discontinuation underlines the need for psychotropic drugs in children with ASD and highlights the need for studies assessing the long-term effects of psychotropic drugs in children with ASD.

## Figures and Tables

**Table 1:** Baseline characteristics of the study population.

	Overall (*N* = 23,935)	2017 Cohort (*N* = 14,210)
**ASD subtype**		
Childhood autism	9303 (38.9%)	6365 (44.8%)
Atypical autism	4314 (18.0%)	2682 (18.9%)
Asperger’s syndrome	6950 (29.0%)	3368 (23.7%)
Other pervasive developmental disorders	3368 (14.1%)	1795 (12.6%)
**Median age at first diagnosis, years (IQR)**		
ASD	9 (6–13)	8 (5–11)
Childhood autism	7 (4–11)	6 (4–9)
Atypical autism	11 (7–14)	9 (6–12)
Asperger’s syndrome	11 (8–14)	10 (8–13)
Other pervasive developmental disorders	10 (7–13)	9 (6–11)
**Sex (%)**		
Male	17,966 (75.1%)	10,731 (75.5%)
Female	5969 (24.9%)	3479 (24.5%)
**Ten most common ICD-10 psychiatric comorbidities (%)**		
Hyperkinetic disorders	6922 (28.9%)	4570 (32.2%)
Reaction to severe stress and adjustment disorders	3340 (14.0%)	1905 (13.4%)
Other behavioural and emotional disorders with onset usually occurring in childhood and adolescence	3201 (13.4%)	1883 (13.3%)
Mild mental retardation	2052 (8.6%)	1147 (8.1%)
Tic disorders	1763 (7.4%)	1172 (8.2%)
Depressive episode	1656 (6.9%)	706 (5.0%)
Mixed specific developmental disorders	1553 (6.5%)	881 (6.2%)
Specific developmental disorders of speech and language	1384 (5.8%)	842 (5.9%)
Other anxiety disorders	1288 (5.4%)	701 (4.9%)
Unspecified mental retardation	1190 (5.0%)	675 (4.8%)
**Selected psychiatric comorbidities (%)**		
ADHD	7293 (30.5%)	4851 (34.1%)
Intellectual disability	3587 (15.0%)	2007 (14.1%)
ADHD and intellectual disability	1092 (4.6%)	656 (4.6%)
Other	13,284 (55.5%)	7566 (53.2%)
None	6484 (27.1%)	3890 (27.4%)

**Table 2:** Early discontinuation and persistence rate of ADHD medication, antidepressants, antipsychotics, and melatonin in children and adolescents 3–17 years old with ASD. Restricted to children initiating treatment between 2010 and 2015. For melatonin, the analysis is restricted to patients initiating treatment 2012–2015.

	Total Number of Patients Initiating Treatment	Fills a Second Prescription within the First 180 Days	Day 180	Day 365	Day 730
**ADHD medication**					
3–5	179	93.30%	82.12%	76.54%	72.63%
6–11	1687	94.01%	81.42%	73.81%	67.10%
12–17	771	94.09%	76.21%	62.00%	55.05%
**Antidepressants**					
3–5	5	-	-	-	-
6–11	315	90.16%	70.16%	61.90%	48.57%
12–17	1336	89.76%	72.22%	62.01%	46.54%
**Antipsychotics**					
3–5	30	-	-	-	-
6–11	599	80.97%	64.77%	55.43%	48.08%
12–17	1087	76.85%	60.44%	52.25%	44.87%
**Melatonin**					
3–5	210	71.90%	61.90%	55.50%	46.41%
6–11	1520	74.52%	62.80%	58.10%	51.52%
12–17	1892	63.98%	50.34%	43.31%	37.85%

**Table 3:** The five most commonly used single drug substances within ADHD medication, antidepressants and antipsychotics in 2017 in children and adolescents 6–17 years old with ASD.

	ADHD Medication		Antidepressants		Antipsychotics	
**6–11 years**	Total number of prescriptions (*n* = 6813)		Total number of prescriptions (*n* = 180)		Total number of prescriptions (*n* = 622)	
	Methylphenidate	4641 (68.1%)	Sertraline	137 (76.1%)	Risperidone	471 (75.7%)
	Atomoxetine	1217 (17.9%)	Fluoxetine	15 (8.3%)	Aripiprazole	126 (20.3%)
	Lisdexamfetamine	848 12.4%)	Imipramine	9 (5.0%)	Levomepromazine	11 (1.8%)
	Dexamfetamine	107 (1.6%)	Venlafaxine	8 (4.4%)	Pimozide	5 (0.8%)
	-		Escitalopram	5 (2.8%)	Chlorprothixene	5 (0.8%)
**12–17 years**	Total number of prescriptions (*n* = 12,832)		Total number of prescriptions (*n* = 2903)		Total number of prescriptions (*n* = 4203)	
	Methylphenidate	7817 (60.9%)	Sertraline	2156 (74.3%)	Risperidone	1808 (43.0%)
	Atomoxetine	3353 (26.1%)	Fluoxetine	587 (20.2%)	Aripiprazole	1328 (31.6%)
	Lisdexamfetamine	1560 (12.2%)	Citalopram	71 (2.4%)	Quetiapine	665 (15.8%)
	Dexamfetamine	92 (0.7%)	Mirtazapine	17 (0.6%)	Olanzapine	157 (3.7%)
	Modafinil	10 (0.1%)	Escitalopram	16 (0.6%)	Chlorprothixene	134 (3.2%)

## Supplementary Materials

The following are available online at http://www.mdpi.com/2077-0383/7/10/339/s1, Table S1: Early discontinuation and persistence rate of ADHD medication, antidepressants, antipsychotics, and melatonin in children and adolescents 3–17 years old with ASD and comorbid ADHD. Restricted to children initiating treatment between 2010 and 2015 with comorbid ADHD. For melatonin, the analysis is restricted to patients initiating treatment 2012–2015, Table S2: Early discontinuation and persistence rate of ADHD medication, antidepressants, and antipsychotics, in children and adolescents 3–17 years old with ASD. Restricted to children initiating treatment between 2000–2015 and 2005–2009, respectively, Table S3: Age at first ASD diagnosis and age at first prescription of ADHD medication, antidepressants, antipsychotics, and melatonin in children and adolescents 6–17 years old with ASD in 2017. Stratified by psychiatric comorbidity, Table S4: Prevalence of the ten most common “other” psychiatric comorbidities in children and adolescents 6–17 years old with ASD and other psychiatric comorbidities in 2017, Figure S1: One-year prevalence proportion for the use of ADHD medication, antidepressants, and antipsychotics from 2010 to 2017 and melatonin from 2012 to 2017 in children and adolescents 3–17 years old with ASD. Stratified by age, Figure S2: Age-stratified prevalence proportion for the use of ADHD medication, antidepressants, antipsychotics, and melatonin for children and adolescents 3–17 years old with ASD. Stratified by year of birth, Figure S3: One-year prevalence proportion for the use (≥1 prescription) of ADHD medication, antidepressants, and antipsychotics from 2010 to 2017 and melatonin from 2012 to 2017 in children and adolescents 3–17 years old with ASD.

## Appendix A

Table showing age of study population from 2010–2017 according to birth year. Bold numbers indicate age range of study population.

**Table:**

Birth Year	Calendar Year							
	2010	2011	2012	2013	2014	2015	2016	2017
1992	**17**	18	19	20	21	22	23	24
1993	**16**	**17**	18	19	20	21	22	23
1995	**15**	**16**	**17**	18	19	20	21	22
1996	**14**	**15**	**16**	**17**	18	19	20	21
1997	**13**	**14**	**15**	**16**	**17**	18	19	20
1998	**12**	**13**	**14**	**15**	**16**	**17**	18	19
1999	**11**	**12**	**13**	**14**	**15**	**16**	**17**	18
2000	**10**	**11**	**12**	**13**	**14**	**15**	**16**	**17**
2001	**9**	**10**	**11**	**12**	**13**	**14**	**15**	**16**
2002	**8**	**9**	**10**	**11**	**12**	**13**	**14**	**15**
2003	**7**	**8**	**9**	**10**	**11**	**12**	**13**	**14**
2004	**6**	**7**	**8**	**9**	**10**	**11**	**12**	**13**
2005	**5**	**6**	**7**	**8**	**9**	**10**	**11**	**12**
2006	**4**	**5**	**6**	**7**	**8**	**9**	**10**	**11**
2007	**3**	**4**	**5**	**6**	**7**	**8**	**9**	**10**
2008	2	**3**	**4**	**5**	**6**	**7**	**8**	**9**
2009	1	2	**3**	**4**	**5**	**6**	**7**	**8**
2010	0	1	2	**3**	**4**	**5**	**6**	**7**
2011		0	1	2	**3**	**4**	**5**	**6**

## Appendix B

Codes used to define psychiatric comorbidities.

**Table:**

Psychiatric Comorbidities	ICD-10 Codes
ADHD	F90, F98.8C
Intellectual disability	F70-F79
Other psychiatric comorbidity	All F-codes with the exception of codes used to define ASD (F84.0, F84.1, F84.5, F84.8, F84.9 *) and codes used to define ADHD and intellectual disability
No psychiatric comorbidity	Absence of ADHD, intellectual disability and other psychiatric comorbidity as defined above

* Not used in the definition of ASD, see Method.
