# Supplementary material for: Use of Psychotropic Drugs among Children and Adolescents with Autism Spectrum Disorders in Denmark: A Nationwide Drug Utilization Study
Source: J Clin Med. 2018 Oct 10;7(10):339. doi: 10.3390/jcm7100339 (PMC6211111; doi:10.3390/jcm7100339)
Supplement: Supplementary file 1 [file jcm-07-00339-s001.zip › Supplementary tables.docx]

**Supplementary tables**

**Table S1**

Early discontinuation and persistence rate of ADHD medication, antidepressants, antipsychotics, and melatonin in children and adolescents 3-17 years old with ASD and comorbid ADHD. Restricted to children initiating treatment between 2010 and 2015 with comorbid ADHD. For melatonin, the analysis is restricted to patients initiating treatment 2012-2015.

|  | **Total number of patients initiating treatment** | **Fills a second prescription within the first 180 days** | **Day 180** | **Day 365** | **Day 730** |
| --- | --- | --- | --- | --- | --- |
| **ADHD medication** |  |  |  |  |  |
| 3-5 | 170 | 94.71% | 83.53% | 78.82% | 75.29% |
| 6-11 | 1520 | 94.99% | 83.40% | 76.80% | 70.48% |
| 12-17 | 576 | 95.36% | 79.29% | 64.61% | 58.69% |
| **Antidepressants** |  |  |  |  |  |
| 3-5 | n<5 | - | - | - | - |
| 6-11 | 133 | 90.98% | 66.92% | 58.65% | 38.35% |
| 12-17 | 289 | 87.59% | 70.07% | 60.78% | 43.87% |
| **Antipsychotics** |  |  |  |  |  |
| 3-5 | 14 | - | - | - | - |
| 6-11 | 361 | 80.33% | 64.54% | 56.51% | 49.31% |
| 12-17 | 354 | 78.07% | 60.82% | 55.91% | 48.43% |
| **Melatonin** |  |  |  |  |  |
| 3-5 | 85 | 77.65% | 69.41% | 65.88% | 51.76% |
| 6-11 | 949 | 75.45% | 64.38% | 60.23% | 52.11% |
| 12-17 | 828 | 65.73% | 53.50% | 45.22% | 43.09% |

**Table S2**

Early discontinuation and persistence rate of ADHD medication, antidepressants, and antipsychotics, in children and adolescents 3-17 years old with ASD. Restricted to children initiating treatment between 2000-2004 and 2005-2009, respectively.

|  |  | **2000-2004** |  |  |  |
| --- | --- | --- | --- | --- | --- |
|  | **Total number of patients initiating treatment** | **Fills a second prescription within the first 180 days** | **Day 180** | **Day 365** | **Day 730** |
| **ADHD medication** |  |  |  |  |  |
| 3-5 | 84 | 80.95% | 66.67% | 58.33% | 57.14% |
| 6-11 | 371 | 90.27% | 75.41% | 65.04% | 58.42% |
| 12-17 | 9 | - | - | - | - |
| **Antidepressants** |  |  |  |  |  |
| 3-5 | 5 | - | - | - | - |
| 6-11 | 69 | 88.41% | 72.46% | 44.93% | 33.33% |
| 12-17 | 7 | - | - | - | - |
| **Antipsychotics** |  |  |  |  |  |
| 3-5 | 18 | - | - | - | - |
| 6-11 | 155 | 84.52% | 70.97% | 62.58% | 57.79% |
| 12-17 | n<5 | - | - | - | - |
|  |  | **2005-2009** |  |  |  |
|  | **Total number of patients initiating treatment** | **Fills a second prescription within the first 180 days** | **Day 180** | **Day 365** | **Day 730** |
| **ADHD medication** |  |  |  |  |  |
| 3-5 | 225 | 88.44% | 78.22% | 71.11% | 72.44% |
| 6-11 | 1381 | 93.04% | 79.86% | 74.33% | 71.04% |
| 12-17 | 390 | 93.04% | 77.06% | 64.74% | 56.55% |
| **Antidepressants** |  |  |  |  |  |
| 3-5 | 8 | - | - | - | - |
| 6-11 | 222 | 81.98% | 61.71% | 54.95% | 47.75% |
| 12-17 | 388 | 91.67% | 78.65% | 64.08% | 48.93% |
| **Antipsychotics** |  |  |  |  |  |
| 3-5 | 30 | - | - | - | - |
| 6-11 | 394 | 85.03% | 72.08% | 62.69% | 56.89% |
| 12-17 | 383 | 78.48% | 63.78% | 55.83% | 53.64% |

**Table S3**

Age at first ASD diagnosis and age at first prescription of ADHD medication, antidepressants, antipsychotics, and melatonin in children and adolescents 6-17 years old with ASD in 2017. Stratified by psychiatric comorbidity.

|  |  | **Comorbidity** | | | | | | |  | |
| --- | --- | --- | --- | --- | --- | --- | --- | --- | --- | --- |
|  | **ASD**  (n=14,210) | **ADHD**  (n=4,851) | **Intellectual disability**  (n=2,007) | | **ADHD and intellectual disability**  (n=656) | | **Other psychiatric comorbidities**  (n=7,566) | | **None**  (n=3,890) | |
| Median age at ASD diagnosis | 8 (5-11) | 8 (6-11) | 5 (4-8) | | 6 (4-9) | | 9 (6-12) | | 7 (5-10) | |
| Age at first prescription of |  | | | | | | | | | |
| ADHD medication | 9 (7-11) | 9 (7-10) | | 8 (6-9) | | 8 (6-9) | | 9 (7-11) | | 9 (7-11) |
| Antidepressants | 14 (12-15) | 13 (11-15) | | 13 (11-16) | | 12 (11-16) | | 14 (12-15) | | 14 (11-15) |
| Antipsychotics | 11 (9-14) | 10 (8-13) | | 10 (8-13) | | 9 (7-11) | | 12 (9-14) | | 11 (9-15) |
| Melatonin | 11 (8-13) | 10 (8-12) | | 9 (7-12) | | 10 (7-12) | | 11 (9-13) | | 10 (8-13) |

**Table S4**

Prevalence of the ten most common “other” psychiatric comorbidities in children and adolescents 6-17 years old with ASD and other psychiatric comorbidities in 2017.

| **Psychiatric comorbidity** | **N (%)** |
| --- | --- |
| F43: Reaction to severe stress, and adjustment disorders | 1952 (25.8%) |
| F98: Other behavioural and emotional disorders with onset usually occurring in childhood and adolescence | 1652 (21.8%) |
| F95: Tic disorders | 1177 (15.6%) |
| F83: Mixed specific developmental disorders | 888 (11.7%) |
| F80: Specific developmental disorders of speech and language | 843 (11.1%) |
| F32: Depressive episode | 728 (9.6%) |
| F41: Other anxiety disorders | 722 (9.5%) |
| F42: Obsessive-compulsive disorder | 595 (7.9%) |
| F93: Emotional disorders with onset specific to childhood | 581 (7.7%) |
| F82: Specific developmental disorder of motor function | 441 (5.8%) |
