# Supplementary material for: Use of Psychotropic Drugs among Children and Adolescents with Autism Spectrum Disorders in Denmark: A Nationwide Drug Utilization Study
Source: J Clin Med. 2018 Oct 10;7(10):339. doi: 10.3390/jcm7100339 (PMC6211111; doi:10.3390/jcm7100339)
Supplement: Supplementary file 1 [file jcm-07-00339-s001.zip › Supplementary tables.pdf]

Supplementary tables

Table S1

Early discontinuation and persistence rate of ADHD medication, antidepressants, antipsychotics, and melatonin in children and adolescents 3-17 years old with ASD and comorbid ADHD. Restricted to children initiating treatment between 2010 and 2015 with comorbid ADHD. For melatonin, the analysis is restricted to patients initiating treatment 2012-2015.

	**Total number of patients initiating treatment**	**Fills a second prescription within the first 180 days**	**Day 180**	**Day 365**	**Day 730**
**ADHD medication**					
3-5	170	94.71%	83.53%	78.82%	75.29%
6-11	1520	94.99%	83.40%	76.80%	70.48%
12-17	576	95.36%	79.29%	64.61%	58.69%
**Antidepressants**					
3-5	n<5	-	-	-	-
6-11	133	90.98%	66.92%	58.65%	38.35%
12-17	289	87.59%	70.07%	60.78%	43.87%
**Antipsychotics**					
3-5	14	-	-	-	-
6-11	361	80.33%	64.54%	56.51%	49.31%
12-17	354	78.07%	60.82%	55.91%	48.43%
**Melatonin**					
3-5	85	77.65%	69.41%	65.88%	51.76%
6-11	949	75.45%	64.38%	60.23%	52.11%
12-17	828	65.73%	53.50%	45.22%	43.09%

Table S2

Early discontinuation and persistence rate of ADHD medication, antidepressants, and antipsychotics, in children and adolescents 3-17 years old with ASD. Restricted to children initiating treatment between 2000-2004 and 2005-2009, respectively.

2000-2004

	**Total number of patients initiating treatment**	**Fills a second prescription within the first 180 days**	**Day 180**	**Day 365**	**Day 730**
**ADHD medication**					
3-5	84	80.95%	66.67%	58.33%	57.14%
6-11	371	90.27%	75.41%	65.04%	58.42%
12-17	9	-	-	-	-
**Antidepressants**					
3-5	5	-	-	-	-
6-11	69	88.41%	72.46%	44.93%	33.33%
12-17	7	-	-	-	-
**Antipsychotics**					
3-5	18	-	-	-	-
6-11	155	84.52%	70.97%	62.58%	57.79%
12-17	n<5	-	-	-	-

2005-2009

	**Total number of patients initiating treatment**	**Fills a second prescription within the first 180 days**	**Day 180**	**Day 365**	**Day 730**
**ADHD medication**					
3-5	225	88.44%	78.22%	71.11%	72.44%

6-11	1381	93.04%	79.86%	74.33%	71.04%
12-17	390	93.04%	77.06%	64.74%	56.55%
**Antidepressants**					
3-5	8	-	-	-	-
6-11	222	81.98%	61.71%	54.95%	47.75%
12-17	388	91.67%	78.65%	64.08%	48.93%
**Antipsychotics**					
3-5	30	-	-	-	-
6-11	394	85.03%	72.08%	62.69%	56.89%
12-17	383	78.48%	63.78%	55.83%	53.64%

Table S3

Age at first ASD diagnosis and age at first prescription of ADHD medication, antidepressants, antipsychotics, and melatonin in children and adolescents 6-17 years old with ASD in 2017. Stratified by psychiatric comorbidity.

	ASD (n=14,210)	Comorbidity				
		**ADHD** (n=4,851)	**Intellectual disability** (n=2,007)	**ADHD and intellectual disability** (n=656)	**Other psychiatric comorbidities** (n=7,566)	**None** (n=3,890)
Median age at ASD diagnosis	8 (5-11)	8 (6-11)	5 (4-8)	6 (4-9)	9 (6-12)	7 (5-10)
Age at first prescription of						
ADHD medication	9 (7-11)	9 (7-10)	8 (6-9)	8 (6-9)	9 (7-11)	9 (7-11)
Antidepressants	14 (12-15)	13 (11-15)	13 (11-16)	12 (11-16)	14 (12-15)	14 (11-15)
Antipsychotics	11 (9-14)	10 (8-13)	10 (8-13)	9 (7-11)	12 (9-14)	11 (9-15)
Melatonin	11 (8-13)	10 (8-12)	9 (7-12)	10 (7-12)	11 (9-13)	10 (8-13)

Table S4

Prevalence of the ten most common “other” psychiatric comorbidities in children and adolescents 6-17 years old with ASD and other psychiatric comorbidities in 2017.

**Psychiatric comorbidity**	**N (%)**
F43: Reaction to severe stress, and adjustment disorders	1952 (25.8%)
F98: Other behavioural and emotional disorders with onset usually occurring in childhood and adolescence	1652 (21.8%)
F95: Tic disorders	1177 (15.6%)
F83: Mixed specific developmental disorders	888 (11.7%)
F80: Specific developmental disorders of speech and language	843 (11.1%)
F32: Depressive episode	728 (9.6%)
F41: Other anxiety disorders	722 (9.5%)
F42: Obsessive-compulsive disorder	595 (7.9%)
F93: Emotional disorders with onset specific to childhood	581 (7.7%)
F82: Specific developmental disorder of motor function	441 (5.8%)
