# Supplementary figures and images for: Use of Psychotropic Drugs among Children and Adolescents with Autism Spectrum Disorders in Denmark: A Nationwide Drug Utilization Study
Source: J Clin Med. 2018 Oct 10;7(10):339. doi: 10.3390/jcm7100339 (PMC6211111; doi:10.3390/jcm7100339)

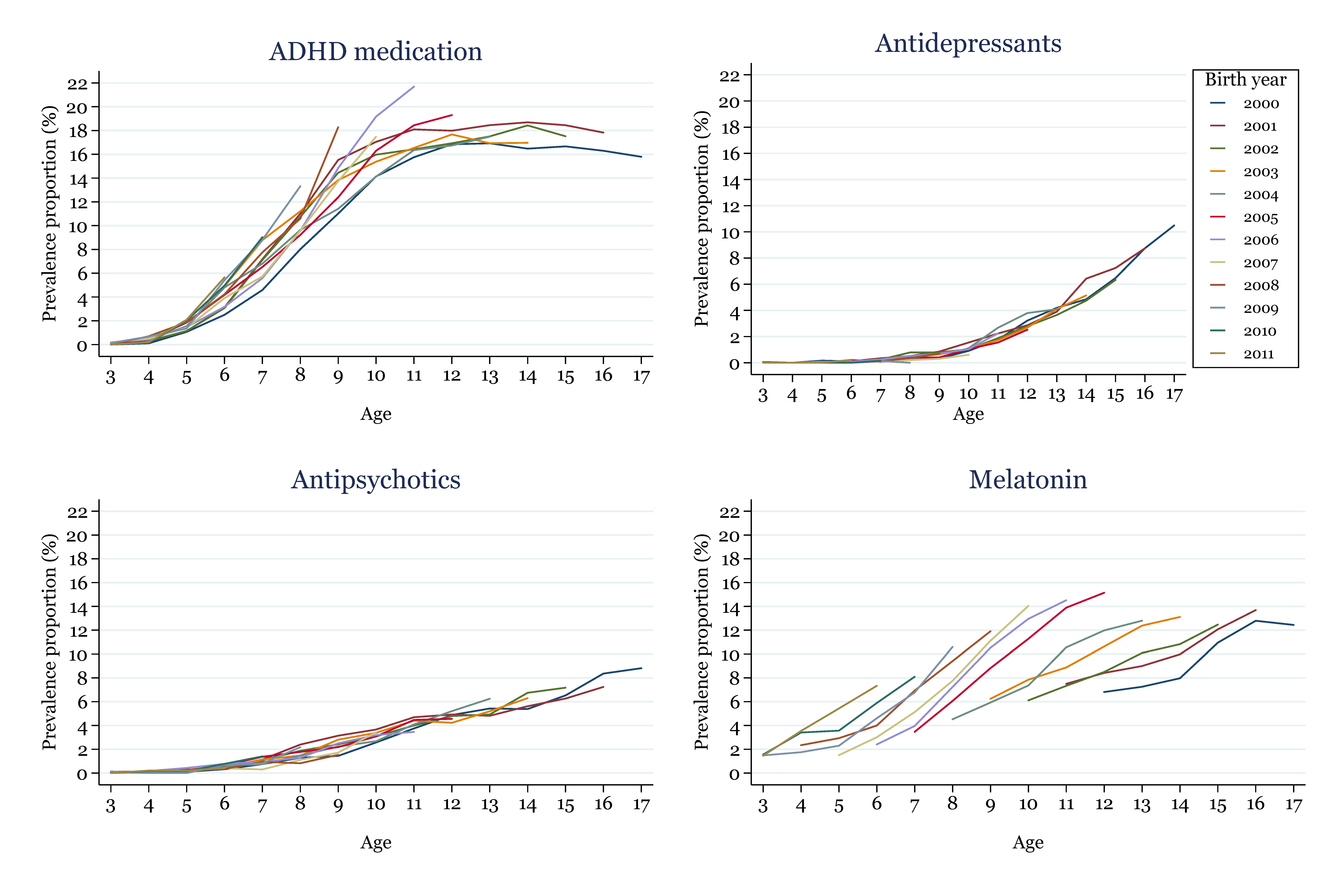

Supplement: Supplementary file 1 [file jcm-07-00339-s001.zip › Figure S2.tif]
